# Lobular capillary hemangioma of the tracheobronchial tree: A case report and literature review

**DOI:** 10.1097/MD.0000000000005499

**Published:** 2016-12-02

**Authors:** Xiaojian Qiu, Zhiwu Dong, Jie Zhang, Jin Yu

**Affiliations:** a Department of Pulmonary Diseases, Beijing Tian Tan Hospital, Capital Medical University, Beijing; b Department of Clinical Chemistry, Jinshan Branch of Shanghai Sixth People's Hospital, Shanghai; c Department of Pathology, Beijing Tian Tan Hospital, Capital Medical University, Beijing, China.

**Keywords:** bronchoscopy, case report, cryotherapy, hemoptysis, lobular capillary hemangioma

## Abstract

**Rationale:**

Lobular capillary hemangioma (LCH) of the tracheobronchial tree is a rare benign tumor, whose characteristics and treatments remain relatively unknown.

**Patient concerns:**

A 39-year-old man with hemoptysis caused by neoplasm in the bronchus intermedius was admitted to our hospital.

**Diagnoses:**

The patient was diagnosed with LCH.

**Interventions:**

The lesions were removed with biopsy forceps, and cryotherapy was performed.

**Outcomes:**

After follow up for more than 2 years, no recurrence was found.

**Lessons:**

Airway LCH can be treated by excisional biopsy, cryotherapy, APC, laser, radiotherapy, and surgery. Cryotherapy is worthy of recommendation.

## 1 Introduction

Lobular capillary hemangioma (LCH) is a benign lesion, which usually affects the skin, mucous membranes of the oral or nasal cavity, and even lips.^[1]^ LCH of the tracheobronchial tree is very rare. Although a few related cases have been reported, none was associated with the bronchus intermedius.^[2–11]^ A case of isolated LCH in the bronchus intermedius was diagnosed with pathologic examination of forceps biopsied specimens. In this case, the patient has been followed up for more than 2 years, and the administered treatments are proved to be very effective. The patient is satisfied.

## 2 Case report

The patient has signed informed consent giving permission to use of his medical data. The protocol for this study was approved by the Institutional Ethics Committee of Beijing Tian Tan Hospital, Capital Medical University. A male patient of 39 years (Han nationality, no occupation) was admitted to our hospital on April 20, 2012. His chief complaint was “cough and expectoration for 2 months, with intermittent blood sputum for 1 month, aggravated for 7 days.” He denied other diseases and a family history of similar symptoms. The patient said he had developed cough after a common cold, with some white sputum; body temperature was up to 38.8°C, with no chills 2 months before. After 10 days of intravenous cephalosporin in a local hospital, his body temperature returned to normal, but cough did not improve significantly. One month before visiting our facility, he presented hemoptysis every 1 to 3 days, and was transferred to Chinese People's Liberation Army General Hospital. Upon bronchoscopic examination, a neoplasm was found in the opening of the bronchus intermedius, and fluorescent bronchoscopy showed a lilaceous structure. Pathological findings were chronic mucosal inflammation in the bronchus intermedius with acute necrosis and formation of granulation tissue, significant partial hyperplasia of the epithelium, and negative periodic acid Schiff (PAS) and acid-fast staining. Tumor cells were not found in the seventh group of mediastinal lymph nodes by EBUS-TBNA. PET scan showed high metabolism in the mediastinum, alerting for lymphoma, but no diagnosis was made.

**Figure 1:**
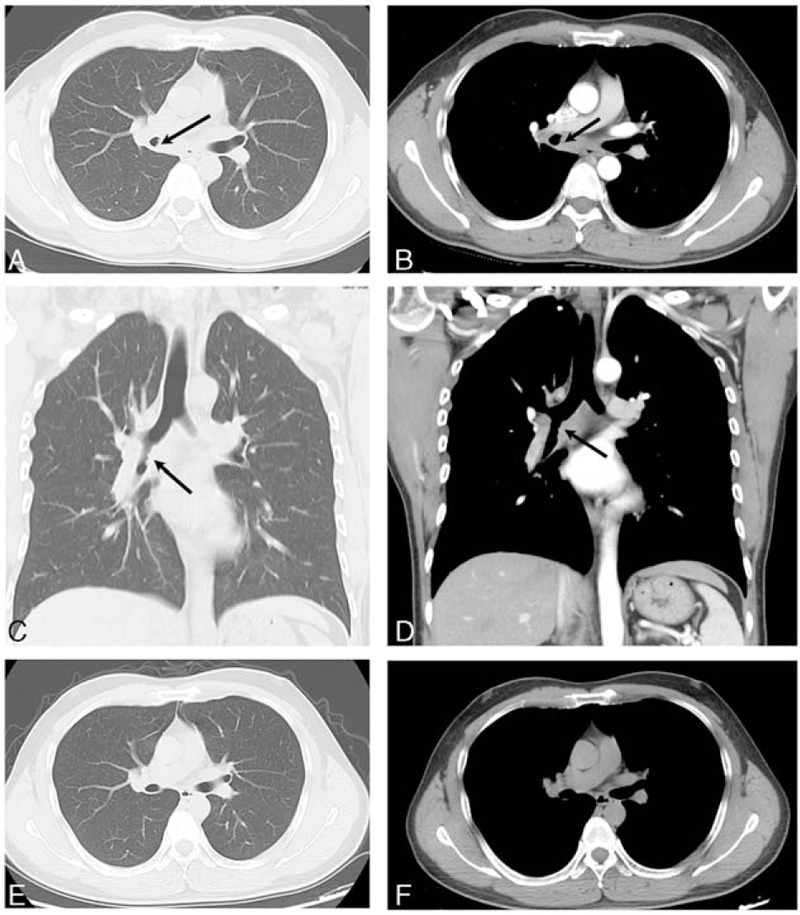
CT scan. (A) Enrollment into hospital; neoplasm in the bronchus intermedius (cross-section, lung window) (April 24, 2012). (B) Cross-section, mediastinal window. (C) Coronal section, lung window. (D) Coronal section, mediastinal window. (E) After 1.5 years; normal bronchus intermedius (cross-section, lung window) (November 5, 2013). (F) Cross-section, mediastinal window (November 5, 2013).

**Figure 2:**
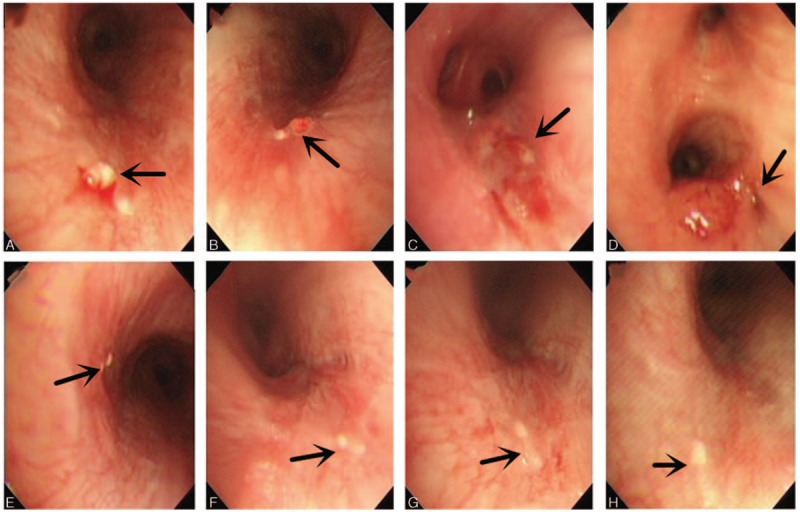
Bronchoscopy. (A) Enrollment into hospital; nodular neoplasm in the bronchus intermedius (April 25, 2012). (B) After 2 weeks; residual root of the neoplasm (May 9, 2012). (C) After 1.5 months; nodular neoplasm recurrence (June 12, 2012). (D) After 4 months; nodular neoplasm recurrence (August 15, 2012). (E) After 6 months; 2 millet size lesions in the bronchus intermedius (November 5, 2012). (F) After 9 months; 2 millet size lesions (January 22, 2013). (G) After 1.5 years; 1 millet size lesion (November 5, 2013). (H) After 2 years; 1 millet size lesion (May 27, 2014).

**Figure 3:**
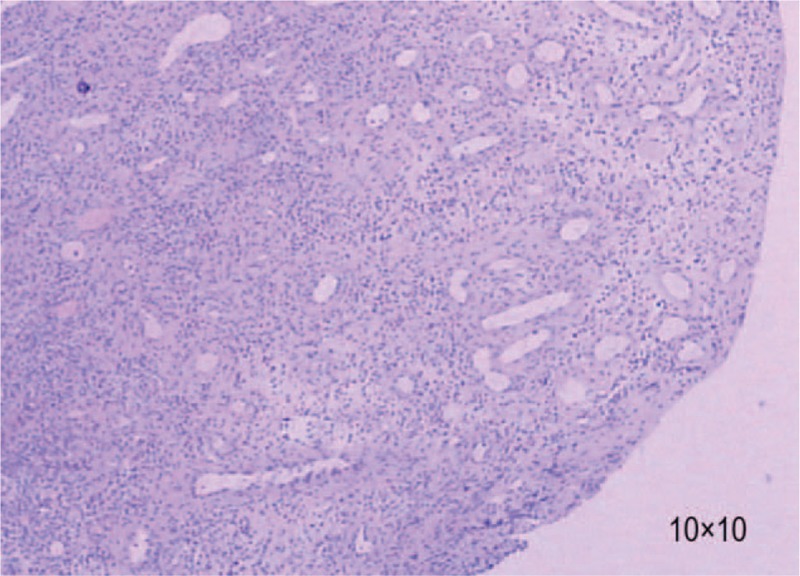
Pathology. Granulation tissue and small cartilage.

Physical examination on admission showed body temperature of 37°C, P of 72/min, R of 18/min, and BP of 120/80 mm Hg. The patient was conscious and active. Lung breath sounds were clear, with no dry or moist rales. Heart borders were normal; heart rate was 72/min. Cardiac rhythm was regular, and no pathological murmurs were observed. The abdomen was flat and soft; liver and spleen were untouched. Lower extremities showed no swelling. Tumor markers, including alpha-fetoprotein (AFP), carcinoembryonic antigen (CEA), carbohydrate antigen 242 (CA242), prostate-specific antigen (PSA), and cytokeratin19 (CK19) were in normal range. Total leukocyte and neutrophil numbers were normal, as well as blood sedimentation. Chest CT showed lesions in the bronchus intermedius, resembling a neoplasm (Fig. 1A–D). Bronchoscopy conducted on April 25, 2012 showed a number of nodular neoplasms obstructing about 1/3 of the lumen; the basement was located on the left wall, with a length of 1.5 cm in the bronchus intermedius (Fig. 2A). We used biopsy forceps to remove most nodules with minimal bleeding, and performed cryotherapy to the tumor basement, with no complications. Pathology of the neoplasm at our hospital showed granulation tissue and small cartilage (Fig. 3). Pathological examination of the neoplasm at Peking Union Medical College Hospital showed a piece of cartilage and multinucleated giant cells in the inflammatory granulation tissue; PAS was positive, while methenamine silver and acid-fast staining were negative. Further pathology expert consultation revealed coated pseudostratified ciliated columnar epithelium and complex stratified squamous epithelium mucosa in chronic inflammation, visible granulation tissue composed of thick walled blood vessels and capillary congestion, vascular interstitial edema, focal epithelioid cell granuloma, and lots of inflammatory cells partially infiltrated. LCH, also known as purulent granuloma, was considered; acid-fast staining was negative. The final diagnosis was LCH.

After 2 weeks (May 9, 2012), the patient returned for reexamination. Bronchoscopy showed the neoplasm was significantly reduced in the bronchus intermedius (Fig. 2B). After 1 month and a half (June 12, 2012), bronchoscopy showed partial recurrence of the neoplasm, and cryotherapy was performed (Fig. 2C). After almost 4 months (August 15, 2012), bronchoscopy showed partial recurrence of the neoplasm, and cryotherapy was performed another time (Fig. 2D). After 6 months (November 5, 2012), bronchoscopy showed 2 small nodules in the bronchus intermedius, and no therapy was administered (Fig. 2E). After 9 months (January 22, 2013), bronchoscopy also showed 2 small nodules in the bronchus intermedius, and no therapy was provided (Fig. 2F). After approximately 1.5 years (November 5, 2013), bronchoscopy showed 1 small nodule in the bronchus intermedius, and no therapy was provided (Fig. 2G). There was no abnormality in the bronchus intermedius by chest CT (Fig. 1E–F). After 2 years (May 27, 2014), bronchoscopy showed 1 small nodule in the bronchus intermedius (Fig. 2H). Currently, the follow-up process is still in progress.

## 3 Discussion

LCH, also called pyogenic granuloma (PG), is a smooth or lobulated capillary hemangioma often appearing in the skin and mucosa, pedunculated or sessile, ranging from a few millimeters to 2.5 cm.^[6,8]^ The mature lesion is a fragile purple red polyp, prone to bleeding and ulceration, often without pedicle after recurrence. LCH is more common in males below 18 years and females of reproductive age.^[9,12,13]^ Pregnancy can stimulate angiogenesis and inhibit its apoptosis.^[14]^

The causes of this disease remain unclear, and may include trauma, drug side effects, hormonal shifts, viral oncogenes, production of angiogenic factors, cytogenetic clonal deletion abnormalities, low-grade local irritation, and traumatic injury.^[10,15]^ Local trauma can lead to the production of angiogenic vascular growth factors, such as vascular endothelial growth factor (VEGF) and decorin; transcription factors (pATF2 and pSTAT3) and signal transduction pathways (MAPK) are overexpressed or activated in LCH, but their exact roles remain unclear.^[11,16]^ LCH is usually accompanied by inflammatory changes, suggesting that a serious imbalance in the site of injury causes granuloma formation. To emphasize these pathological features, the term LCH was recently coined.^[1]^ LCH might have been caused in this patient by low-grade local irritation following infection.

This tumor can occur on skin surface and mucosal membranes. Fechner et al^[1]^ retrospectively analyzed 639 cases of vascular lesions located in the mouth, upper respiratory tract, throat, and trachea, and found 73 LCH cases, with all lesions found in the nasal cavity or oral mucosa. One of the 62 cases with vascular lesions in the trachea or larynx showed granulation tissue, but with no evidence of LCH pathology. LCH in the gastrointestinal tract was also reported.^[17]^ LCH lesions are very rare in the airway, with only 11 cases reported in the trachea and left main bronchus.^[2–11]^Table 1 summarizes all reported LCH cases, including the current one.

**Table 1:**
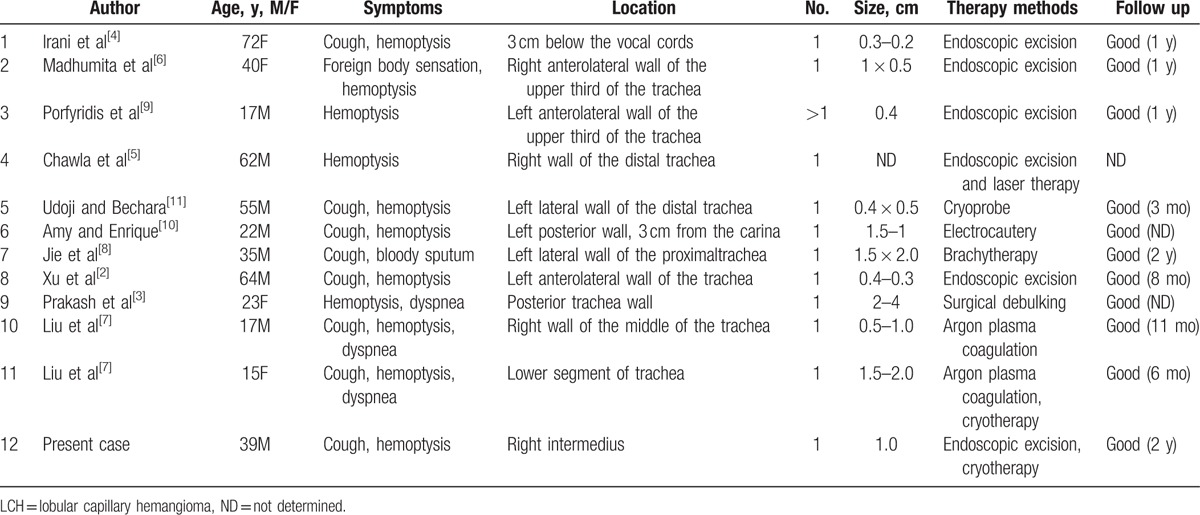
Summary of LCH case reports.

The main LCH symptoms are bleeding and airway obstruction, with no pain.^[8]^

This patient had the symptoms of cough and blood sputum. Histological examination revealed numerous capillaries arranged in a lobular pattern, occasionally surrounded by a heaped-up collar of normal tissue. These lesions, however, were altered by secondary inflammatory changes, thus forming granulomas.^[8]^ Acute or chronic inflammatory cells are scattered around the lesion, and mostly found on the surface. The invading pathogenic microorganisms usually appear on the ulcer surface. A fibromyxoid background between the capillary lobules is also characteristic of LCH. Diagnosis relies on chest CT and bronchoscopy.^[8]^ Chest CT can identify the occupying lesions, while bronchoscopy biopsy achieves final diagnosis. Differential diagnosis of airway LCH includes granulation tissue, angioendothelioma, angiosarcoma, tufted hemangioma, intravascular angiomatosis, and Kaposi sarcoma.^[5,17]^ Diagnosis in this patient depended on pathological findings.

Treatments of LCH of the skin or mucous membrane include laser, liquid nitrogen freezing, microwave irradiation, brachytherapy, intralesional injection of ethanol or corticosteroids, and sodium tetradecyl sulfate sclerotherapy.^[8,15]^ Similar methods are used for tracheobronchial LCH. Three cases were treated with cryotherapy,^[2,7,11]^ after which the lesions located in the distal trachea in 1 case were not recurrent after 3 months.^[11]^ One case was treated with biopsy forceps for lesions located in the trachea, and no recurrence was found in 8 months.^[2]^ Another case was treated with surgery,^[3]^ while 2 patients were treated with APC.^[7]^ A patient with lesions located in the distal trachea was treated with laser, and neodymium-doped yttrium aluminum garnet photocoagulation and mechanical debulking were used.^[5]^ The lesion in another case was located in the distal left main bronchi, and not controlled after 9 repetitions of tracheal endoscopic electrocautery and APC. Afterwards, the patient was treated 3 times with high-dose rate endotracheal brachytherapy, at a total dose of 18 Gy (6 Gy/fraction, 2 times/wk). The patient was followed up for more than 2 years without recurrence.^[8]^ At present, there is no comparison between various treatment methods for LCH of the tracheobronchial tree. All methods generally achieve good therapeutic effects, but long-term observation is still needed. For this case, we first used biopsy forceps to remove the neoplasm, then performed cryotherapy for the residual lesions. The latter were gradually reduced, finally becoming a small nodule. The patient has been followed up for more than 2 years, with no recurrence. Therefore, for treating LCH of the tracheobronchial tree, cryotherapy can be performed under local anesthesia, with good tolerance, less damage, good curative outcome and few side effects, and is worthy of recommendation. However, optimizing the treatment of tracheobronchial LCH still requires a large number of controlled clinical trials.

A recurrence rate of 16% was reported for oral LCH; the causes were incomplete excision, failure to eradicate the etiological factors, or reinjury of the area.^[18]^ Reports of recurrence in the tracheobronchial tree are rather scarce.

## 4 Conclusion

LCH of the tracheobronchial tree is a rare disease; clinical manifestations are hemoptysis and dyspnea. It is diagnosed mainly by bronchoscopic biopsy, and can be treated by excisional biopsy, cryotherapy, APC, laser, radiotherapy, and surgery. Among them, cryotherapy can be performed under local anesthesia, with good tolerance, less damage, good curative outcome, and few side effects, and is worthy of recommendation.
